# Deep learning-based dose prediction for prostate cancer with empty bladder protocol: a framework for efficient and personalized radiotherapy planning

**DOI:** 10.3389/fonc.2025.1690416

**Published:** 2025-12-17

**Authors:** Byongsu Choi, Deepak K. Shrestha, Albert Attia, Brad J. Stish, James Leenstra, Jean Claude Rwigema, Jiansen Ma, Sung Uk Lee, Jong Hwi Jeong, Jongeun Kim, JeongHeon Kim, Chris Beltran, Justin C. Park

**Affiliations:** 1 Department of Radiation Oncology, Mayo Clinic, Jacksonville, FL, United States; 2 Department of Radiation Oncology, Mayo Clinic, Rochester, MN, United States; 3 Department of Radiation Oncology, Mayo Clinic, Northfield, MN, United States; 4 Department of Radiation Oncology, Mayo Clinic, Scottsdale, AZ, United States; 5 Department of Quantitative Health Sciences, Mayo Clinic, Rochester, MN, United States; 6 Proton Therapy Center, National Cancer Center Korea, Goyang-si, Republic of Korea

**Keywords:** deep learning, dose prediction, empty bladder, particle therapy, prostate, radiation therapy

## Abstract

Radiation therapy (RT) is a cornerstone in the management of localized and locally advanced prostate cancer, traditionally delivered with a full bladder (FB) protocol to reduce radiation exposure to surrounding organs. However, consistent bladder filling is difficult to maintain, leading to workflow delays, anatomical inconsistencies, and variable toxicity outcomes. Recent evidence, including the ongoing RELIEF trial at Mayo Clinic, suggests that an empty bladder (EB) protocol provides comparable toxicity outcomes to FB while improving patient comfort and treatment consistency. To address the increased anatomical variability associated with EB protocols, we developed a deep learning (DL)-based dose prediction model tailored to EB patients. A conditional generative adversarial network (cGAN) with a modified 3D U-Net architecture was trained on 90 FB cases and fine-tuned on 20 EB cases stratified into stereotactic body radiotherapy (SBRT) and intensity-modulated radiotherapy (IMRT). Model performance was evaluated against clinical manual plans using mean absolute percentage error (MAPE) and dose-volume histogram (DVH) metrics. The EB Fine-tuning model(SBRT/IMRT) achieved superior accuracy compared with the general FB-trained model, with an average MAPE of 3.53 ± 0.40% versus 4.87 ± 0.86%. DVH analyses demonstrated improved agreement with manual plans for planning target volumes and organs at risk, with discrepancies consistently within 2.5 Gy or 3%. These results demonstrate that fine-tuning with EB-specific data enhances prediction accuracy and clinical relevance of the DL-based model. The proposed framework supports efficient EB treatment planning, provides reference dose distributions for quality assurance, and offers educational value to clinicians adopting EB protocols. By combining automation with clinical applicability, this approach facilitates broader adoption of EB radiotherapy in prostate cancer while improving workflow reproducibility and patient-centered care.

## 1 Introduction

High-quality radiation treatment planning for prostate cancer is a complex and time-intensive process, often requiring multiple iterations between clinicians and dosimetrists to achieve clinical-quality plans. Traditionally, radiation therapy (RT) for prostate cancer, including the initial computed tomography (CT) simulation, is conducted with a full bladder (FB) protocol. This approach aims to reduce radiation exposure to surrounding organs, such as the bladder, small bowel, and rectum (1–5). However, maintaining consistent bladder filling during CT simulation and daily treatments can be challenging, resulting in inter-fractional variations in patient anatomy and potentially prolonged treatment times (6, 7). Such variations can hinder reproducibility in patient anatomy, potentially affecting treatment accuracy. Studies have explored alternative approaches to mitigate these challenges. For instance, Burns et al. reported no significant differences in bladder volume consistency when a more comfortable bladder-filling regimen was used, compared to the traditional FB protocol (8). Similarly, Moieenko et al. found no correlation between bladder filling and prostate location shifts, nor an impact on Planning Target Volume (PTV) coverage (9). Furthermore, recent findings indicate that treating prostate cancer patients with an empty bladder (EB) protocol results in non-inferior organ toxicity compared to the standard FB technique (10, 11). At Mayo Clinic, the ongoing RELIEF (Randomized Evaluation of the Impact of Empty Versus Full Bladder) trial compares EB and FB protocols, evaluating both patient experiences and radiation-induced toxicity outcomes.

Despite ongoing technological progress in radiation therapy, treatment planning for prostate cancer remains a resource-intensive process that heavily relies on manual input and iterative adjustments between clinicians and dosimetrists. This manual approach not only limits operational efficiency but also hampers the adaptability required for patient-specific anatomical variations. These limitations become more pronounced with the implementation of emerging protocols such as the empty bladder (EB) technique, which deviates from the conventional full bladder (FB) approach and introduces increased anatomical variability—particularly in the bladder, bowel, and rectal positions. Such variability can complicate dose optimization, increase inter-observer variability, and reduce consistency in treatment outcomes.

Given the growing interest in EB protocols due to their potential advantages in patient comfort and treatment reproducibility, there is a critical need for robust, automated solutions that can support their broader clinical adoption. Specifically, data-driven dose prediction models capable of generating accurate, anatomy-informed treatment plans offer a scalable path forward. These models can serve as clinical references for EB cases, reduce planning time, and facilitate more standardized, reproducible care across institutions. In this context, deep learning presents a powerful tool to address these challenges by utilizing prior data to guide individualized and protocol-specific radiotherapy planning.

The application of deep learning (DL) in empty-bladder (EB) radiation therapy planning holds significant promise (12–16). DL models excel at analyzing large datasets and generating precise dose distributions, making them well-suited to address the unique challenges associated with EB protocols. Unlike full-bladder (FB) approaches, EB planning introduces greater anatomical variability, particularly in surrounding organs, which can complicate manual planning (17–22). Training DL models on EB-specific datasets can enable the development of data-driven frameworks capable of producing accurate, anatomy-informed dose distributions. These models not only reduce reliance on manual planning but also help overcome the learning curve for clinicians unfamiliar with EB protocols.

Additionally, DL-based approaches can account for patient-specific anatomical variations, enabling the creation of personalized treatment plans aligned with clinical goals (23). Beyond clinical utility, these models can provide educational value offering visual and quantitative insights that support both planners and clinicians. Integrating DL into the EB planning workflow has the potential to enhance efficiency, improve plan quality and encourage broader clinical adoption of this emerging protocol.

In summary, the aim of this study was to develop and evaluate a deep learning–based dose prediction model specifically adapted for the empty-bladder protocol. We demonstrate that EB-specific fine-tuning substantially improves dose accuracy over a general FB-trained model, reducing total MAPE from 4.87% to 3.53% and improving DVH agreement across PTV and OARs. These findings highlight the feasibility and clinical relevance of protocol-tailored DL models for EB radiotherapy planning. In this study, we developed a deep learning (DL)-based model to predict 3D dose distributions for patients assigned to the empty bladder (EB) arm of the RELIEF trial. Given the ongoing patient enrollment, we initially trained a General model using a dataset of 90 prostate cancer patients treated with a full bladder (FB) protocol at Mayo Clinic Florida (MCF). To enhance the model’s specificity for EB cases, we fine-tuned it using data from 20 EB patients enrolled in the RELIEF trial across all three Mayo Clinic sites. The proposed workflow supports seamless integration into clinical practice. After the patient is randomized to the EB arm prior to CT simulation, Organs at risk (OARs) are segmented using a commercial AI-based auto contouring software, followed by manual target delineation by the radiation oncologist. The DICOM CT and RT structure set are then input into the dose prediction model, which generates a 3D dose distribution overlaid on the patient’s CT image and structure set. This output serves as a reference for medical dosimetrists, guiding the development of an optimal clinical plan and identifying potential challenges. Simultaneously, the initial predicted dose distribution functions as a pre-planning quality assurance (QA) tool, offering radiation oncologists a preview of a dosimetrically achievable final plan.

## 2 Materials and methods

### 2.1 Model

In the conventional deep learning framework, let f be a vector of functions that are learned using the original prostate patient dataset which is a subset generated from the total prostate patient dataset as ( $X_{FB},Y_{FB})\subset \left(X,Y\right)$. Here $X_{FB}$ represents input images from the FB prostate patient dataset and $Y_{FB}$ denotes the corresponding dose maps. If f maps $X_{FB}$ to $Y_{FB}$, then the goal is to find the parameterized inverse mapping function $f_{\theta }$ by optimizing the loss function (Equation 1):

(1) $$\hat{\theta }\;=\underset{\theta }{\text{argmin}}\left\{\frac{1}{N}\sum_{\left(x,y\right)\in \left(X_{FB},Y_{FB}\right)}E\left(f_{\theta }\left(x\right),y\right)\right\}$$

A primary limitation of this framework arises when the dataset fails to adequately capture the true data manifold, leading to poor generalization of the model to unseen data points. Although conventional techniques such as weight decay and dropout have been employed to address this issue, achieving robust generalization remains inherently challenging when the model does not effectively capture the latent space representation of unobserved data.

To overcome this limitation, we developed a fine-tuned model for the empty bladder (EB) dataset using a two-step approach. In the first step, the model was trained on the FB dataset of size N. In the second step, the model was fine-tuned using the EB dataset of size M*M*, creating a refined model specifically tailored to the EB patient group. The EB dataset, ( $X_{EB},Y_{EB})\subset \left(X,Y\right)$, was derived by selecting prostate cancer patients treated under the EB protocol.

The two-stage optimization process is defined as follows

(2) $${\hat{\theta }}_{first}=\underset{\theta }{\text{argmin}}\left\{\frac{1}{N}\sum_{\left(x,y\right)\in \left(X_{FB},Y_{FB}\right)}E\left(f_{\theta }\left(x\right),y\right)\right\}$$

(3) $${\hat{\theta }}_{second_{SBRT}}=\underset{\theta_{SBRT}}{\text{argmin}}\left\{\frac{1}{M}\sum_{\left(x,y\right)\in \left(X_{EB},Y_{EB}\right)}E\left(f_{\theta }\left(x\right),y\right)\right\}$$

(4) $${\hat{\theta }}_{\sec ond_{IMRT}}=\underset{\theta_{IMRT}}{\text{argmin}}\left\{\frac{1}{M}\sum_{\left(x,y\right)\in \left(X_{EB},Y_{EB}\right)}E\left(f_{\theta }\left(x\right),y\right)\right\}$$

For clarity, the fine-tuning was performed separately for SBRT ( $\theta_{SBRT}$) and IMRT ( $\theta_{IMRT}$) shown in Equations 3 and 4, resulting in two modality-specific EB models. The proposed method builds upon the knowledge learned from the General FB dataset to refine the model for the specific EB patient group. In this two-step process, the first step involves training a General model (Equation 2) on the broader prostate dataset ( $X_{FB},Y_{FB}$), followed by fine-tuning the model in the second step using the specific EB dataset ( $X_{EB},Y_{EB}$). This approach ensures that the model retains a comprehensive understanding of General prostate patient anatomy while adapting to the unique characteristics of EB patients.

To enhance the fine-tuning process, we employed data augmentation techniques, such as random rotations, translations, and scaling, to increase the variability and diversity of the training data. This augmentation helps the model better learn the anatomical differences and dose distribution patterns unique to EB patients. Additionally, we adopted a conditional generative adversarial network (cGAN) architecture, where the generator predicts dose distributions, and the discriminator evaluates the realism of the generated outputs compared to the ground truth dose maps. This adversarial framework ensures that the model not only minimizes pixel-wise errors but also generates clinically realistic dose distributions.

Through this methodology, the fine-tuned model is designed to achieve improved performance in predicting dose distributions for EB prostate patients. By effectively capturing the unique anatomical and clinical features of EB patients while utilizing the generalized knowledge from the FB dataset, the model addresses the challenges of data variability and ensures improved accuracy and clinical relevance in dose prediction.

### 2.2 Overview of framework

**Figure 1:**
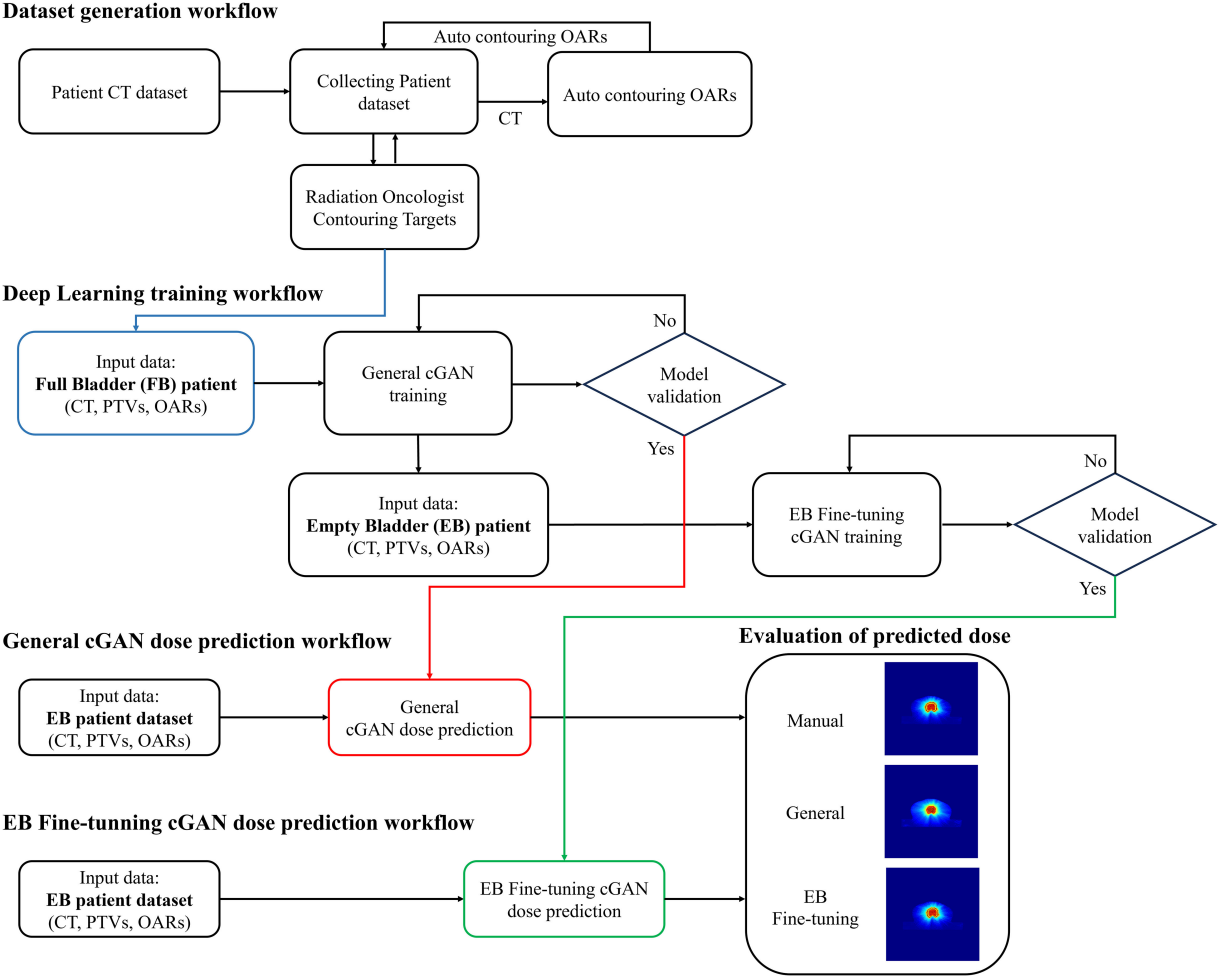
Workflow of the proposed framework for dose prediction and evaluation. The figure outlines the four key stages of the framework: data generation workflow, deep learning training workflow, dose prediction models and evaluation of predicted dose.

**Figure 1** illustrates the proposed framework, which comprises four main components: data generation workflow, deep learning training workflow, dose prediction workflow, evaluation of predicted dose and additionally providing experiential insights into EB-specific dose distributions for radiation oncologists. The framework begins with an automated dataset generation process. Patient CT datasets were used for the initial organ-at-risk (OAR) contours. These contours are then reviewed and refined by a radiation oncologist, who also delineates the target volumes. The finalized structure sets and CT images are categorized into full bladder (FB) and empty bladder (EB) groups for model training and evaluation. Through this workflow, the predicted dose distributions offer clinicians a clear and intuitive understanding of what dosimetrically optimized EB treatment plans look like, thereby supporting both planning and education for EB protocol adoption.

Using the FB patient dataset, a conditional Generative Adversarial Network (cGAN) is trained to develop a General model capable of predicting dose distributions based on the CT images, target structures, and OARs. This General model is then fine-tuned using a smaller EB patient dataset to create the EB-specific cGAN model for SBRT patients and IMRT patients. For dose prediction, the fine-tuned model is applied exclusively to test datasets consisting of EB patients.

In the final step, the predicted doses from both the General and fine-tuned models are evaluated using metrics such as Mean Absolute Percentage Error (MAPE) and Dose-Volume Histograms (DVH), reflecting clinical priorities. The EB-specific model, which demonstrates superior performance, is used to generate dose distributions that can assist radiation oncologists in planning and serve as an educational tool for consistently managing the EB cases in the future.

**Figure 2:**
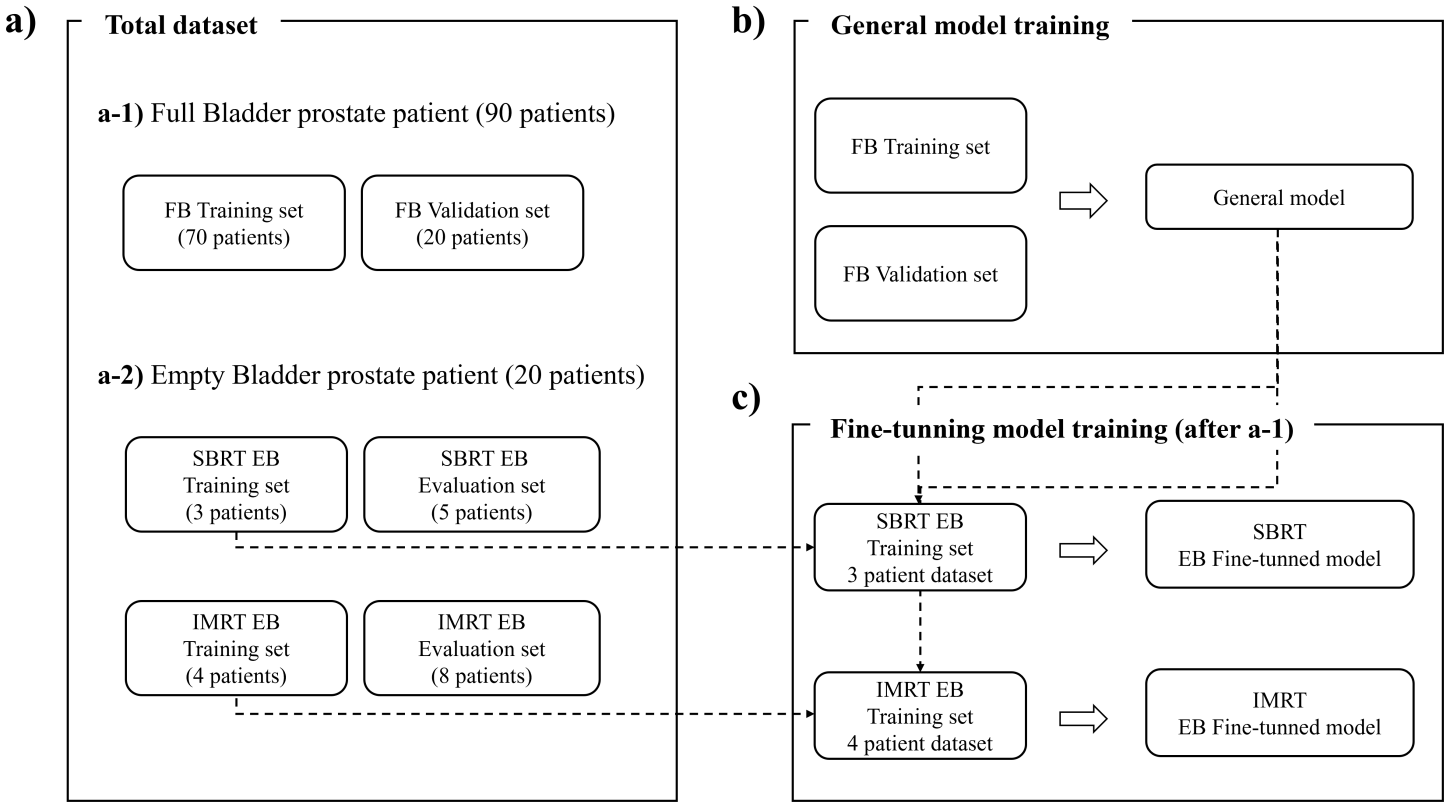
Dataset structure for dose prediction workflow. The total dataset **(a)** includes FB patients (a-1, FB Training & FB Validation set) and EB patients (a-2, n=20). General model training trained with FB patient dataset using a-1 **(b)**. Fine-tuning **(c)** uses the EB dataset (a-2) to optimize predictions, while evaluation (a-2, EB Evaluation set) validates the model’s performance.

The dataset used in this study is illustrated in **Figure 2** and divided into three main subsets: the total dataset, the General training dataset, and the fine-tuning dataset. The total dataset comprises 110 prostate cancer patients, including 90 full-bladder (FB) cases and 20 empty-bladder (EB) cases. The 90 FB cases consisted of 24 SBRT and 66 IMRT plans. Because the FB dataset was IMRT-dominant (66/24), the General model naturally learns a dose-pattern prior that is more characteristic of IMRT plans. No modality weighting or balanced sampling strategy was applied during FB training, which may bias the learned mid-dose modulation toward IMRT-like falloff. This imbalance is later corrected through separate modality-specific EB fine-tuning (SBRT-only, IMRT-only), which adapts the model to the appropriate prescription characteristics. The EB dataset is further categorized into 8 stereotactic body radiotherapy (SBRT) cases and 12 intensity-modulated radiation therapy (IMRT) cases.

Initially, the General model is trained using 90 FB cases. Subsequently, two EB-specific models are fine-tuned: one using the 8 SBRT EB cases and the other using the 12 IMRT EB cases. From the EB dataset, 7 patients (3 SBRT and 4 IMRT) are allocated for fine-tuning, while the remaining 13 patients (5 SBRT and 8 IMRT) are reserved for evaluation.

The training process follows a two-step approach. First, a General model is trained on the FB dataset, as described in Equation 1. Then, this model is fine-tuned separately for the SBRT and IMRT subgroups using the EB dataset, as outlined in Equation 2. This approach enables the development of EB-specific models optimized for accurately predicting dose distributions tailored to each treatment modality. Training on the larger FB dataset ensures that the General model effectively learns prostate anatomy and dose distribution patterns. Fine-tuning then adapts this knowledge to account for the anatomical variations unique to EB patients. Following training, both the General model and the EB-specific models are evaluated on the held-out EB test cases (5 SBRT and 8 IMRT) to assess their predictive performance and clinical relevance.

### 2.3 Network architecture

**Figure 3:**
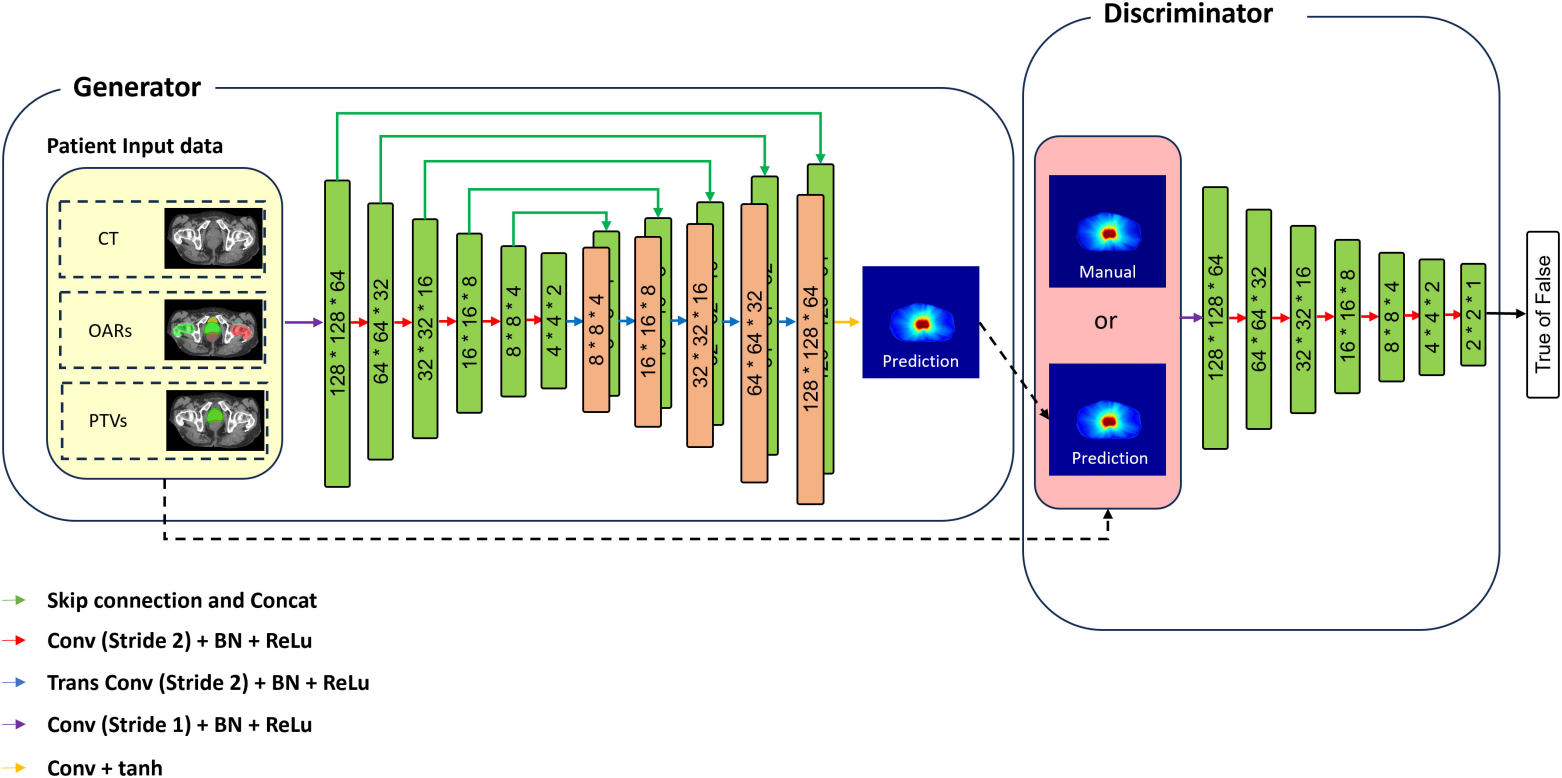
cGAN architecture for dose prediction. The generator, a modified 3D U-Net, processes CT images, OARs, and PTVs to predict 3D dose distributions using convolutional layers, batch normalization, Leaky ReLU activation, transposed convolutions, hyperbolic tangent activation, and skip connections. The discriminator compares manual and predicted dose maps to improve the generator’s accuracy through adversarial learning.

This network is designed to establish a robust voxel-level correlation between multichannel data—comprising anatomical features such as CT scans, planning target volumes (PTVs), and OARs—and the corresponding radiation dose distributions. Utilizing a 3D cGAN framework, the architecture integrates two advanced deep learning methodologies: adversarial learning and full-scale feature fusion within an encoder-decoder structure (**Figure 3**). The architecture consists of two components: a generator that predicts realistic dose distributions conditioned on the input data, and a discriminator that evaluates the authenticity of the generated dose maps by distinguishing them from real clinical dose maps. This adversarial interaction enhances the model’s ability to produce high-quality, patient-specific dose predictions. Additionally, since the GAN model focuses on bladder-specific dose distributions, incorporating conditional information about OARs and target structures related to the bladder enhances the input for both the generator and discriminator. This approach enriches the learning process, culminating in an optimized cGAN framework tailored for precise and reliable dose predictions.

The generator employs a modified multi-channel 3D-UNet architecture with an encoder-decoder design. The encoder extracts hierarchical spatial features using down sampling blocks, each consisting of 3D convolutional layers (4×4×4 kernels) with batch normalization and leaky ReLU activations. The decoder restores the spatial resolution using transposed convolutions with the same kernel size while incorporating skip connections from the encoder to preserve fine-grained details of the input data (CT, OARs, and PTVs). The final layer applies a hyperbolic tangent (tanh) activation function to generate the dose distribution.

The discriminator in the proposed cGAN framework is a critical component for assessing the accuracy of the generated dose maps. It utilizes a hierarchical convolutional architecture to progressively down sample the input data, producing a probability map. In this map, values closer to 1 represent real, manual dose maps, while values closer to 0 indicate synthetic dose maps generated by the model.

The overall objective of the proposed cGAN framework combines adversarial loss and pixel-wise loss, each contributing to different aspects of model optimization (Equation 5):

(5) $$L=\underset{G}{\min}\;\underset{D}{\max}L_{cGAN}\;(G,D)+\lambda L_{pixel}\left(G\right)$$

where $L_{cGAN}\left(G,D\right)$ represents the adversarial loss, which ensures the realism of the generated dose maps. The $L_{pixel}\left(G\right)$ denotes the pixel-wise loss, which guarantees voxel-level accuracy in the predictions. The weight factor λ is used to balance the contributions of the adversarial and pixel-wise losses during optimization. In this study, the weight value λ was set to 100, which was determined empirically to strike a balance between ensuring the generator produces realistic outputs (adversarial loss) and maintaining voxel-level accuracy (pixel-wise loss).

The adversarial loss, $L_{cGAN}\left(G,D\right)$ encourages the generator to create dose maps indistinguishable from real ones while the discriminator learns to differentiate between real and synthetic maps (Equation 6):

(6) $$L_{cGAN}=E_{x,y∼P_{data}}\left[\log D\left(x,y\right)\right]+E_{z_{∼}p_{z}\left(z\right),y∼p\left(y\right)}\left[\log\left(1-D\left(G(\left(z|y\right),y\right)\right)\right]$$

where x represents the real, manual dose map, while y corresponds to the conditional input data, such as CT images, organs at risk (OARs), and planning target volumes (PTVs). z denotes the latent noise vector. The D(x,y): indicates the discriminator’s probability that x is a real dose map, conditioned on y. Lastly, G((z|y) refers to the dose map generated by the generator, conditioned on  y.

The pixel-wise loss, $L_{pixel}\left(G\right)$, minimizes voxel-level differences between the ground truth dose map ( x) and the predicted dose map G(z|y) (Equation 7):

(7) $$L_{pixel}=E_{x,y∼P_{data},z∼P_{z}\left(z\right)}{\left[\left\|x-G\left(z|y\right)\right\|\right]}_{1}$$

The L1​-norm (Mean Absolute Error, MAE) is employed to prioritize sharp and detailed dose distributions, ensuring alignment between the generated and real dose maps. The combined adversarial and pixel-wise losses work synergistically to refine the generator’s ability to produce dose maps that are both clinically realistic and highly accurate. The adversarial loss drives the generator to improve realism, while the pixel-wise loss ensures precise dose distributions at the voxel level.

The cGAN was implemented using TensorFlow v2.8, an open-source deep learning library optimized for scalable and efficient model training. Python was used as the programming language, due to its flexibility and compatibility with TensorFlow. The training and evaluation processes were performed on a single NVIDIA TITAN RTX GPU, equipped with 24 GB of VRAM. The General model was trained using a dataset of 90 FB prostate patient cases. This stage required 250 epochs to ensure adequate convergence of both the generator and discriminator networks. Using a subset of 7 EB patient cases, the fine-tuning stage refined the General model to adapt to EB-specific anatomical variations. This stage required 150 epochs, as the model was able to utilize the pre-trained weights from the General training phase. The batch size was set to 4, optimized to utilize the available GPU memory while maintaining computational efficiency. The Adam optimizer was employed with a learning rate of 1e-4, chosen to ensure smooth convergence during training.

As shown in **Supplementary Figure A**, both generator and discriminator loss remain stable until approximately 250 epochs; beyond this point, noticeable oscillation and divergence appear, indicating adversarial imbalance and overfitting. Similarly, **Supplementary Figure B** shows that validation loss begins to rise after ~150 epochs even as training loss continues decreasing, supporting 150 epochs as the empirically justified early-stopping point for EB fine-tuning. This instability is characteristic of adversarial training dynamics; after extensive iterations, the generator begins overfitting local dose textures while the discriminator becomes overconfident, leading to oscillatory gradients. Beyond 250/150 epochs, we also observed increased variance in generator loss, consistent with mode collapse tendencies described in GAN literature. These behaviors justify selecting 250 (General) and 150 (EB fine-tuning) epochs as optimal stopping points to avoid adversarial imbalance and over-smoothing of dose gradients. The corresponding loss curves for the General model (**Supplementary Figure A**) and EB fine-tuning (**Supplementary Figure B**) illustrate the stabilization phase and subsequent adversarial oscillation that informed the selected epoch thresholds.

### 2.4 Data acquisition

Following retrospective Institutional Review Board (IRB) approval, 90 patients previously treated with radiotherapy (RT) for prostate cancer at Mayo Clinic Florida (MCF) were selected for this study. Additionally, data from 20 patients enrolled in the RELIEF trial were obtained from all three Mayo Clinic sites. All patients in this combined cohort received treatment using either IMRT or SBRT techniques. For each patient, the available CT images, RT Dose, RT Structure, and RT Plan were collected. All clinical treatment plans were generated using Eclipse v15.6 (Varian Medical Systems, Palo Alto, CA, USA) with the Anisotropic Analytical Algorithm (AAA) and a 2.5 mm dose calculation grid. These details are included to enhance reproducibility and facilitate implementation of similar workflows in other clinical centers. Dose-volume histogram (DVH) data for targets and organs at risk (OARs) were extracted for patients included in the training set.

### 2.5 Evaluation

After training both the General and Fine-tuning models, dose distributions were predicted for the evaluation set, which included 5 patients. The accuracy of each model’s dose prediction was evaluated using the MAPE, which quantifies the relative error between the predicted and ground truth doses. The calculation, as shown in Equation 8, normalizes the dose difference to the patient’s highest prescribed dose. For example, if a patient had prescribed dose levels of 56, 60, and 70 Gy, the dose difference was divided by 70 Gy, the highest dose. All evaluations were performed in Gray (Gy) to maintain consistency.

(8) $$MAPE\;\left(\%\right)=\frac{100}{n}\sum_{i=1}^{n}\left|\frac{D_{predicted_{i}}-D_{ground\;truth_{i}}}{D_{highest\;dose}}\right|$$

Additionally, DVH metrics were analyzed to evaluate the dosimetric coverage of the PTV and OARs. Key priorities in prostate treatment, such as PTV Dmax and Dmean for targets, as well as V70Gy and V65Gy for OARs, were assessed to quantify the clinical relevance of the predicted dose distributions. These evaluations provided a comprehensive analysis of the accuracy of the models and their ability to generate clinically acceptable dose plans. Furthermore, qualitative comparisons between the predicted and actual dose distributions were conducted to further assess the performance and clinical applicability of the models. To aid visualization, a heatmap was generated, enabling a clear comparison of the dose distributions from the manual plan, EB Fine-tuning model(SBRT/IMRT), and General model.

## 3 Results

**Figure 4:**
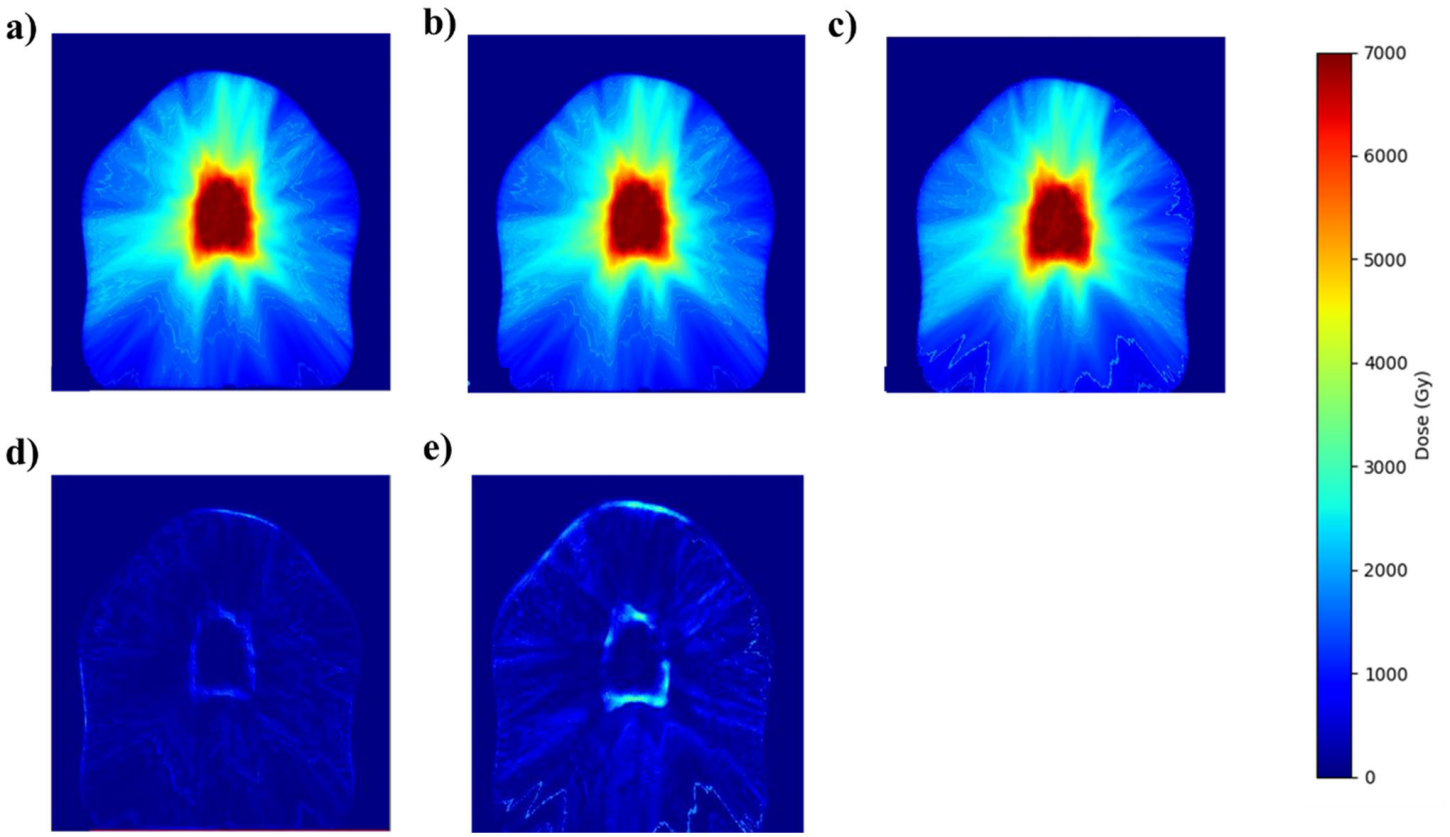
Dose comparison between manual planning **(a)**, the SBRT EB Fine-tuning model(SBRT/IMRT) **(b)**, and the General model **(c)**. Panels **(d)** and **(e)** show the difference maps between manual planning and the EB Fine-tuning model(SBRT/IMRT) **(a, b)**, and manual planning and the General model **(a–c)**, respectively.

**Figure 5:**
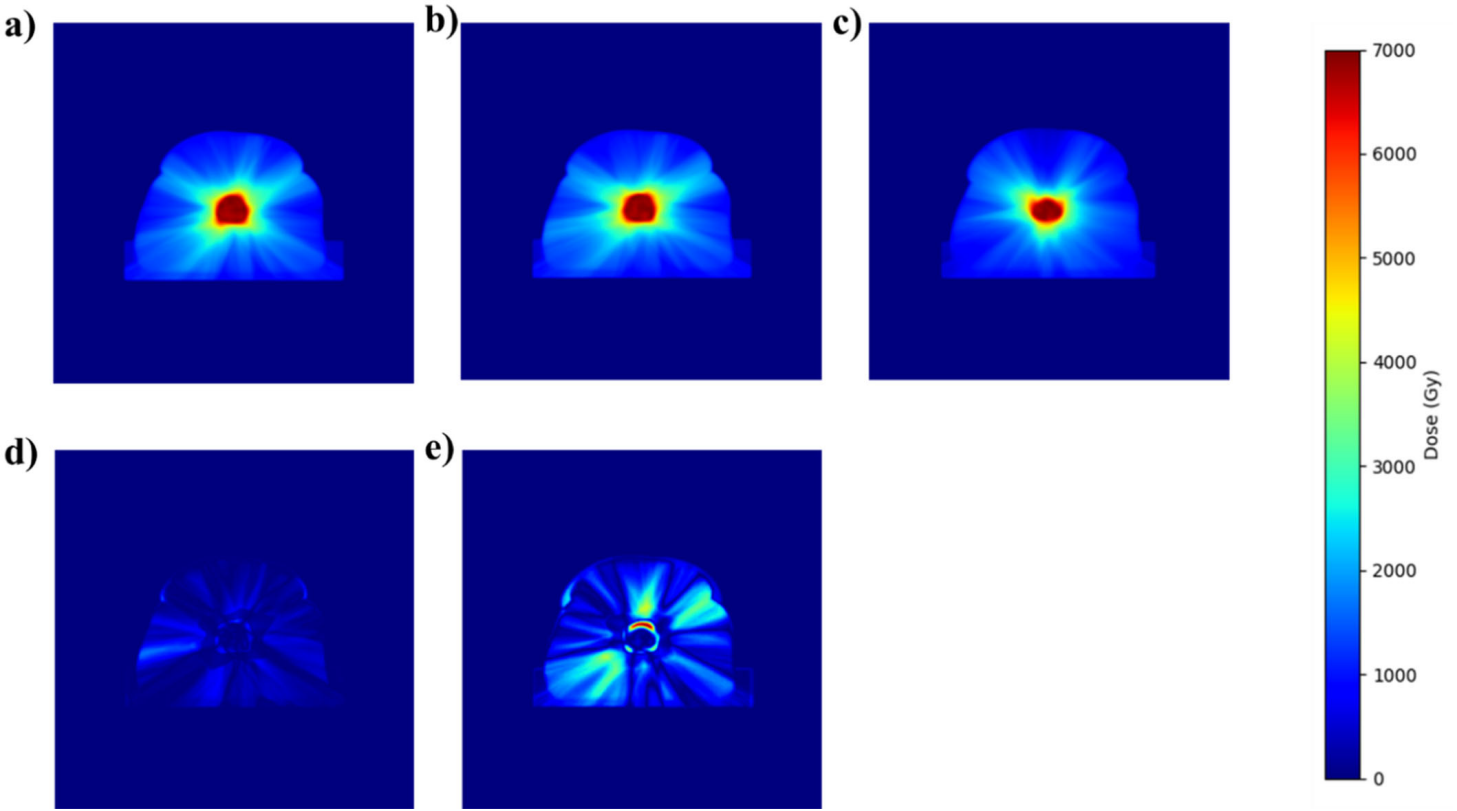
Dose distribution comparisons for Manual planning **(a)**, the IMRT EB Fine-tuning model(SBRT/IMRT) **(b)**, and the General model **(c)**. Panels **(d, e)** show the corresponding difference maps, illustrating deviations between Manual planning and the IMRT EB Fine-tuning model(SBRT/IMRT) **(a, b)**, and between Manual planning and the General model **(a–c)**, respectively.

**Figures 4**, **5** demonstrate the adaptability and accuracy of the EB fine-tuning approach across two distinct treatment modalities—SBRT and IMRT. **Figure 4** compares dose distributions from Manual planning (a), the SBRT EB Fine-tuning model(SBRT/IMRT) (b), and the General model (c) in the top row. The second row illustrates the corresponding difference maps: the left panel shows the discrepancy between the Manual planning and the SBRT EB Fine-tuning model(SBRT/IMRT) (a-b), while the right panel highlights differences between the Manual planning and the General model (a-c). The color scale represents dose intensity in Gy, with red indicating the highest dose (70 Gy) and blue representing the lowest (0 Gy). On the other hand, **Figure 5** illustrates dose distributions obtained through Manual planning (a), the IMRT EB Fine-tuning model(SBRT/IMRT) (b), and the General model (c) in the top row. The second row presents different maps: the left panel depicts the differences between Manual planning and the IMRT EB Fine-tuning model(SBRT/IMRT) (a-b), whereas the right panel shows discrepancies between Manual planning and the General model (a-c). The color scale on the right denotes dose intensity levels, with red corresponding to higher doses (70 Gy) and blue indicating lower doses (0 Gy).

The manual dose distribution acts as the ground truth, exhibiting precise coverage of the target volume. Predictions by the General model display broader dose dispersion, resulting in notable inaccuracies in high-dose areas. The SBRT Fine-tuning model, however, provides a dose distribution closely matching the manual plan, effectively limiting dose exposure beyond the target. The difference maps explicitly demonstrate reduced errors and improved conformity achieved by the SBRT Fine-tuning model relative to the General model.

The manually planned dose distribution, serving as the benchmark, accurately targets the prescribed area. Predictions by the General model exhibit evident deviations, particularly in the high-dose regions, leading to excessive dose spread. In contrast, the IMRT Fine-tuning model closely replicates the manual dose distribution, maintaining accurate high dose targeting and minimizing unintended dose exposure to adjacent structures. The difference maps emphasize the IMRT Fine-tuning model’s significantly reduced errors compared to the General model, highlighting its enhanced clinical applicability and accuracy.

In both SBRT and IMRT cases, the manual dose distribution serves as the ground truth, demonstrating precise high-dose coverage of the target volume. Predictions from the General model exhibit broader dose dispersion and noticeable inaccuracies in high-dose regions, resulting in excessive dose spread beyond the intended treatment area. In contrast, the EB Fine-tuning model(SBRT/IMRT)s for both SBRT and IMRT closely replicate the manual dose distributions, effectively maintaining dose conformity and minimizing unnecessary exposure to surrounding healthy tissues. The corresponding difference maps further highlight the superior performance of the fine-tuning models, showing reduced errors and improved alignment with clinical standards. These findings underscore the enhanced accuracy and clinical applicability of the EB-specific models across multiple treatment modalities.

A paired Wilcoxon signed-rank test confirmed that the EB Fine-tuning model(SBRT/IMRT) achieved significantly lower total MAPE than the General model (p = 0.003). To support visual comparisons in **Figures 4**, **5**, all heatmaps were normalized to the prescription dose of 70 Gy, and difference maps represent voxel-wise absolute deviations in Gy.

**Figure 6:**
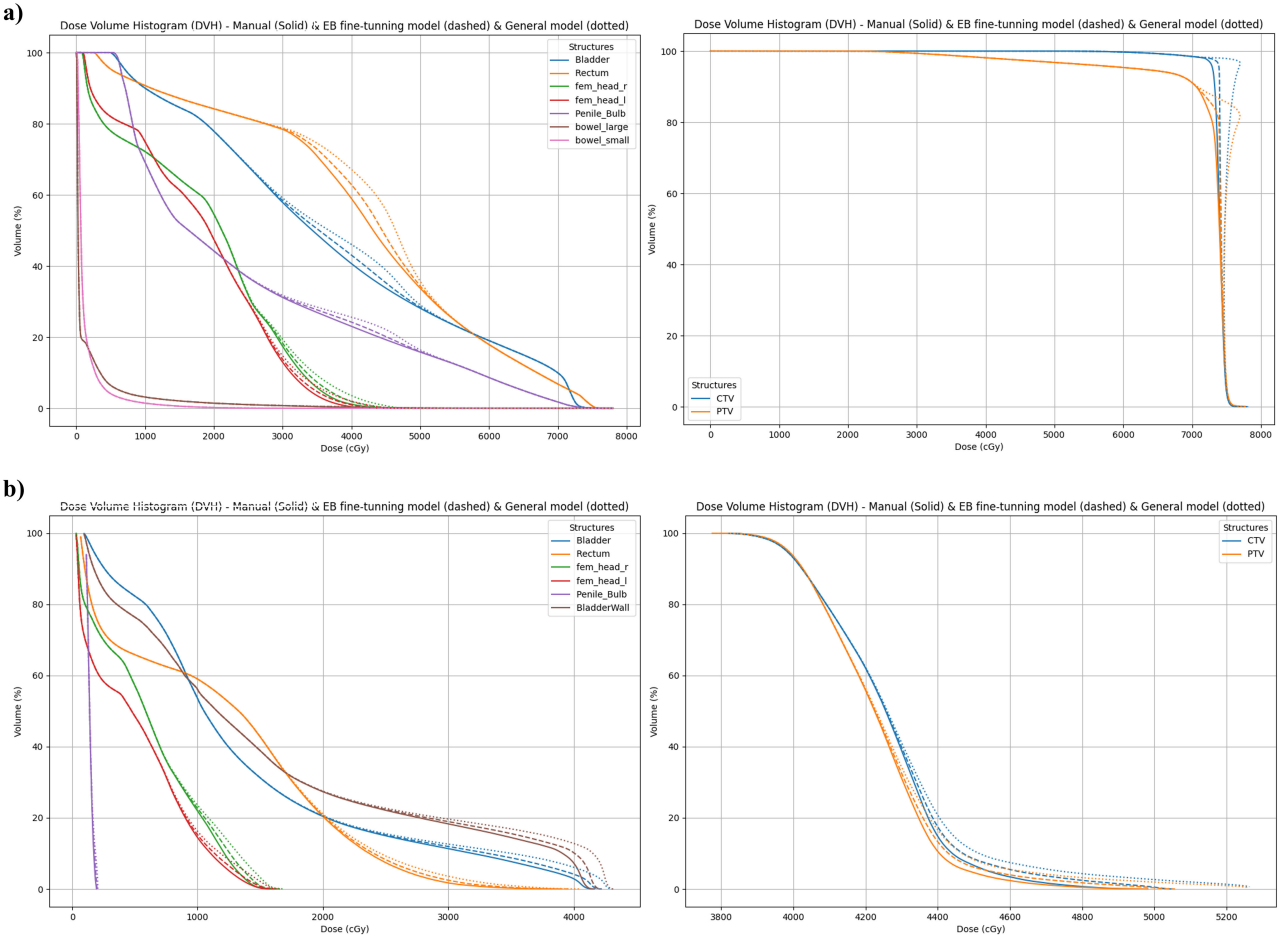
DVH comparisons between the Manual plan (solid lines), the EB Fine-tuning model(SBRT/IMRT) (dotted lines), and the General model (dashed lines). DVHs were generated for the following regions of interest (ROIs): PTV, CTV, rectum, penile bulb, right femoral head (fem_head_r), left femoral head (fem_head_l), large bowel, bladder, and bladder wall. Panel **(a)** shows results from the SBRT patient dataset, and panel **(b)** shows results from the IMRT patient dataset.

A DVH comparison for the same case obtained with different plans is shown in **Figure 6**. When comparing the Manual plan to the EB Fine-tuning model(SBRT/IMRT) and the General model, the EB Fine-tuning model(SBRT/IMRT) demonstrated results that were closely aligned with the Manual plan, while the General model exhibited significant deviations.

For the PTV, the following metrics were analyzed: Dmean, D99%, V107%, and Dmax. The EB Fine-tuning model(SBRT/IMRT) showed differences of (2.12± 0.67) Gy compared to (4.12 ± 2.28) Gy for the General model in Dmean. For D99%, the differences were (2.49 ± 0.54) % for the EB Fine-tuning model(SBRT/IMRT) and (5.57 ± 3.09) % for the General model. Dmax differences were (1.67 ± 2.48) Gy and (3.92 ± 1.99) Gy for the EB Fine-tuning and General models, respectively.

**Figure 7:**
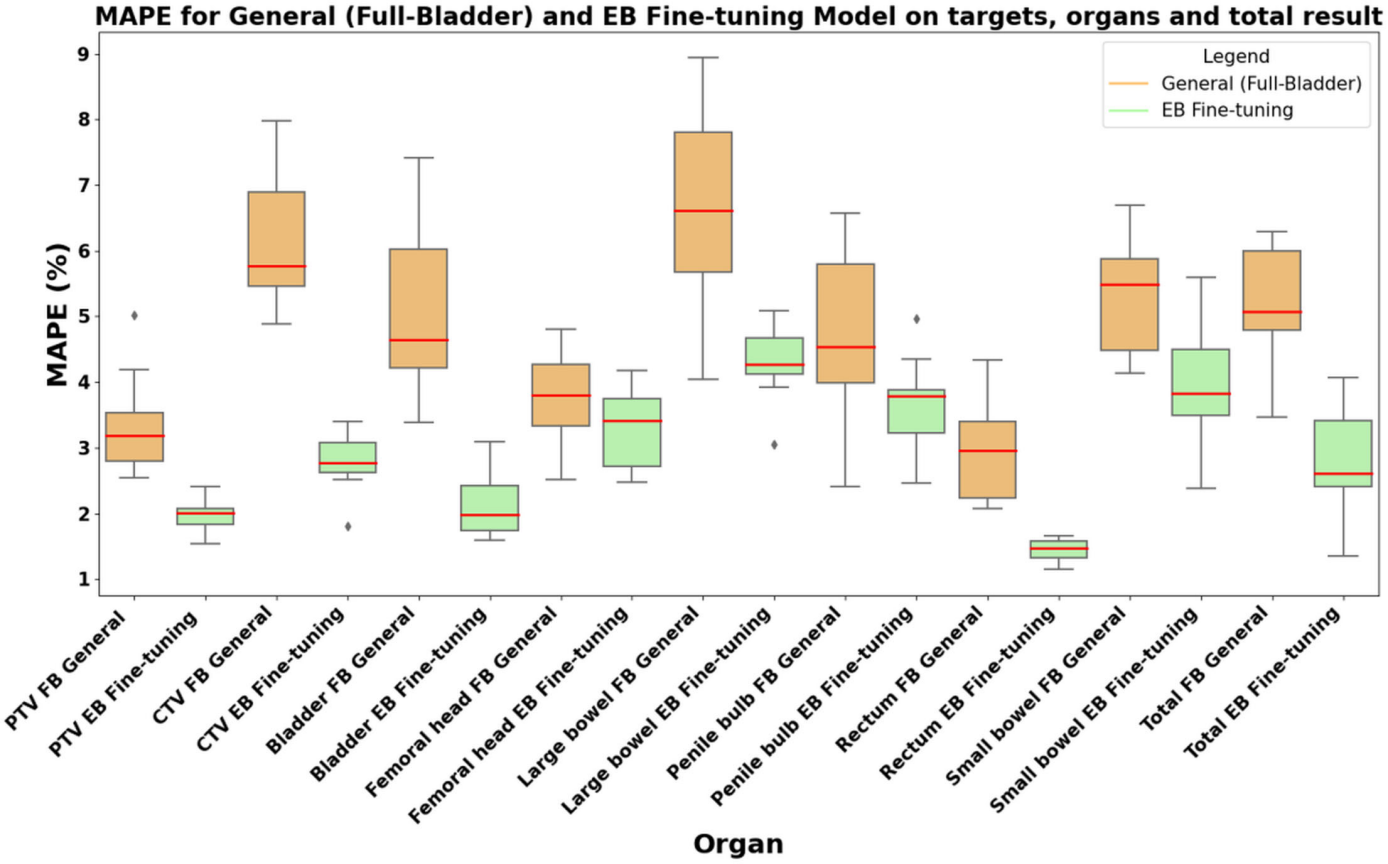
Mean Absolute Percentage Error (MAPE) comparison between the General model (Full-Bladder) and the Fine-Tuning model (Empty-Bladder) across targets, organs, and the total dose distribution.

**Figure 7** illustrates the total MAPE of the General model and the Fine-tuning model using 8 Regions of Interests (ROIs) and the total dose distribution results, which contains the bladder, CTV, femoral head, large bowel, penile bulb, PTV, rectum and small bowel. As shown, the EB Fine-tuning model(SBRT/IMRT) outperformed the General model in most cases, particularly for the bladder, large bowel, CTV, and PTV. The total average MAPE for the General model was (4.87 ± 0.86) %, whereas the EB Fine-tuning model(SBRT/IMRT) achieved a significantly lower total average of (3.53 ± 0.40) %. Additionally, the EB Fine-tuning model(SBRT/IMRT) demonstrated consistently lower MAPE values across all individual ROIs, emphasizing its superior performance. Notably, substantial improvements were observed for critical structures such as the bladder, rectum, PTV, and CTV, showcasing the EB Fine-tuning model(SBRT/IMRT)’s strength in accurately predicting dose distributions for priority organs and targets.

For the organs-at-risk (OARs), metrics V35Gy, V30Gy, and V25Gy were analyzed for the SBRT cohort, while V70Gy, V65Gy, and V60Gy were evaluated for patients treated with IMRT. For the rectum, the EB Fine-tuning model(SBRT/IMRT) yielded absolute differences of (1.73 ± 0.72) Gy, (2.13 ± 1.60) Gy, and (2.22 ± 0.15) Gy for V35Gy, V30Gy, and V25Gy, respectively. The General model showed greater discrepancies of (3.32 ± 1.01) Gy, (4.57 ± 2.89) Gy, and (4.84 ± 4.15) Gy for the same metrics. Similarly, for the bladder, the EB Fine-tuning model(SBRT/IMRT) achieved absolute differences of (2.35 ± 1.59) Gy (V35Gy), (2.90 ± 1.98) Gy (V30Gy), and (2.35 ± 0.95) Gy (V25Gy). The General model exhibited larger differences of (5.04 ± 3.56) Gy (V35Gy), (4.88 ± 4.66) Gy (V30Gy), and (4.78 ± 3.24) Gy (V25Gy). All values were computed using the absolute differences between predicted and ground-truth dose values.

Overall, the PTV results for the EB Fine-tuning model(SBRT/IMRT) were within 2.5 Gy, while the errors for the OARs were lower, staying within 3%. The average errors of the EB Fine-tuning model(SBRT/IMRT) were consistently smaller than those of the General model, with significant improvements observed for the PTV and Bladder metrics. **Tables 1**, **2** summarize the DVH metrics for the PTV, Rectum, and Bladder of SBRT and IMRT treated patients. The EB Fine-tuning model(SBRT/IMRT) significantly outperformed the General model by underestimating the DVH metric discrepancies. **Figure 6** illustrates boxplots comparing these differences, showing that the EB Fine-tuning model(SBRT/IMRT) produced results closer to the Manual plan, with deviations closer to zero compared to the General model.

**Table 1:** Summary of average DVH metrics and comparison results for the PTV, Rectum, and Bladder in SBRT plans across the five evaluated patients.

ROI	DVH Metric	Manual	EB Fine tuning	General	|Manual – EB Fine-tuning|	|Manual – General|
PTV	Dmean	42.25 ± 1.56 Gy	40.13 ± 2.23 Gy	38.13 ± 3.84 Gy	2.12± 0.67	4.12 ± 2.28
	D99%	37.74 ± 1.59 Gy	35.25 ± 2.13 Gy	32.17 ± 4.68 Gy	2.49 ± 0.54	5.57 ± 3.09
	Dmax	49.81 ± 1.23 Gy	47.96 ± 1.47 Gy	45.89 ± 3.22 Gy	1.67 ± 2.48	3.92 ± 1.99
Rectum	V35Gy	0.46 ± 1.33%	2.19 ± 2.05%	3.78 ± 2.34%	1.73 ± 0.72	3.32 ± 1.01
	V30Gy	2.55 ± 0.86%	4.68 ± 2.46%	7.12 ± 3.75%	2.13 ± 1.60	4.57 ± 2.89
	V25Gy	5.3 ± 2.24%	7.52 ± 2.39%	10.14 ± 6.39%	2.22 ± 0.15	4.84 ± 4.15
Bladder	V35Gy	5.13 ± 2.59%	7.48 ± 4.18%	10.17 ± 6.15%	2.35 ± 1.59	5.04 ± 3.56
	V30Gy	10.36 ± 3.15%	13.56 ± 5.13%	15.24 ± 7.81%	3.20 ± 1.98	4.88 ± 4.66
	V25Gy	11.83 ± 5.23%	14.18 ± 6.18%	16.61 ± 8.47%	2.35 ± 0.95	4.78 ± 3.24

**Table 2:** Overview of average DVH metrics and comparative results for the PTV, Rectum, and Bladder in IMRT treatments of eight patients.

ROI	DVH Metric	Manual	EB Fine tuning	General	|Manual – EB Fine-tuning|	|Manual – General|
PTV	Dmean	72.34 ± 0.65 Gy	70.13 ± 0.85 Gy	67.47 ± 2.12 Gy	2.21 ± 0.02	4.87 ± 1.47
	D99%	61.02 ± 3.65 Gy	58.37 ± 4.75 Gy	55.57 ± 6.21 Gy	2.65 ± 1.10	5.45 ± 2.56
	Dmax	75.42 ± 1.49 Gy	73.44 ± 1.94 Gy	71.46 ± 3.53 Gy	1.98 ± 0.45	3.96 ± 2.04
Rectum	V60Gy	1.91 ± 1.68%	2.35 ± 2.18%	3.48 ± 2.86%	0.44 ± 0.50	1.57 ± 1.18
	V55Gy	4.69 ± 3.17%	5.77 ± 4.12%	8.09 ± 5.39%	1.07 ± 0.95	3.39 ± 2.22
	V50Gy	7.80 ± 4.90%	9.22 ± 5.37%	12.95 ± 7.31%	1.42 ± 0.47	5.15 ± 2.41
Bladder	V60Gy	4.55 ± 2.55%	5.65 ± 3.32%	7.93 ± 4.23%	1.10 ± 0.77	3.38 ± 1.68
	V55Gy	8.75 ± 3.38%	9.67 ± 4.39%	11.49 ± 5.76%	0.92 ± 1.01	2.74 ± 2.38
	V50Gy	11.73 ± 4.15%	13.86 ± 5.39%	15.33 ± 7.08%	2.13 ± 1.24	3.59 ± 2.93

## 4 Discussion

This study presents a comprehensive workflow that encompasses the development of a deep learning (DL)-based dose prediction model, specifically tailored for prostate cancer patients treated under an empty bladder (EB) protocol. Traditionally, radiation therapy for prostate cancer has been predominantly conducted using a full bladder (FB) protocol to minimize radiation exposure to surrounding organs. However, recent research suggests that EB protocols can achieve comparable clinical outcomes to FB protocols while potentially simplifying treatment preparation and improving patient comfort (6, 10, 24). Studies have indicated that EB filling protocols can be effectively implemented in external beam radiotherapy for localized prostate cancer, with no significant differences in treatment outcomes compared to FB protocols.

In our study, we identified significant differences in the dosimetric parameters of the bladder, rectum, and PTV between EB and FB plans across both SBRT and IMRT modalities. These variations underscore the need for EB-specific dose prediction models to address the unique anatomical characteristics associated with this protocol. Our conditional generative adversarial network (cGAN)-based framework successfully fine-tuned a model for EB patients, demonstrating significant improvements in dose prediction accuracy compared to a General FB model. The EB Fine-tuning model(SBRT/IMRT) achieved a reduced total average Mean Absolute Percentage Error (MAPE) of 3.53 ± 0.40%, compared to 4.87 ± 0.86% for the General model. Dose-Volume Histogram (DVH) analyses further validated the model’s clinical utility, with the fine-tuned model showing improved alignment with Manual plans, particularly for critical organs such as the bladder, rectum, and PTV.

Although the numerical difference in total MAPE (3.53% vs. 4.87%) appears modest, this corresponds to 1.0–2.5 Gy deviations in regions of steep dose gradients, which are clinically meaningful for PTV coverage and OAR sparing in SBRT and IMRT. The mid-dose region (approximately 35–40 Gy) exhibited the largest discrepancies due to a combination of architectural and physical factors. First, U-Net skip-connections inherently smooth intermediate-intensity features, reducing the sharpness of the dose-shoulder region where SBRT/IMRT plans often contain complex modulation. Second, the MAE-dominant supervision (λ=100) penalizes voxel-level deviations but does not explicitly constrain DVH-critical dose-shoulder accuracy, causing conservative underestimation. These interactions amplify errors specifically in the mid-dose band. Even small reductions in mid-dose error can alter threshold-based metrics such as V35Gy or V70Gy, reinforcing the clinical relevance of the EB fine-tuning improvements. In addition, the uniformity region of the PTV (D80–100%) exhibited lower accuracy compared to the high-dose core (e.g., D0.03cc). This discrepancy is primarily driven by architectural averaging effects in the U-Net decoder: skip-connection blending reduces the sharpness of the dose plateau, leading to smoothing across the uniform dose layers. Furthermore, the MAE-dominated loss function provides weaker supervision in the dose-shoulder and plateau ranges, prioritizing the minimization of high-dose voxel errors rather than enforcing uniformity across the mid-to-high dose band. These combined factors explain why D80–100% showed larger deviations despite accurate hotspot reproduction. This mid-dose deviation (35–40 Gy) arises from two technical factors. First, the U-Net encoder–decoder architecture intrinsically smooths sharp SBRT dose fall-offs, causing the dose-shoulder region to be underestimated. Second, the MAE-dominant loss function prioritizes voxel-wise accuracy in the high-dose core over preserving the complexity of mid-dose gradients. As a result, the model tends to produce conservative dose estimates specifically in this 30–40 Gy transition band, consistent with the dose-bin error trends in **Supplementary Figure C**. Importantly, the predicted dose distributions generated by this model can serve as more than just a validation tool—they function as actionable clinical references during the treatment planning process. By providing a dosimetrically sound and anatomically informed benchmark early in the planning workflow, these predictions help guide dosimetrists in crafting optimal clinical plans while offering radiation oncologists a reliable previsualization of achievable dose coverage. This dual role supports both quality assurance and decision-making, enabling faster convergence toward clinically acceptable plans, reducing inter-observer variability, and facilitating more standardized implementation of EB protocols. As a result, the model enhances not only the precision but also the efficiency and reproducibility of personalized radiotherapy planning in the EB setting.

In addition to its clinical utility, this framework holds significant educational value. As EB protocols are still emerging in clinical practice, many institutions lack standardized workflows or sufficient reference plans tailored to this approach. The model’s ability to generate anatomically and dosimetrically accurate dose distributions provides clinicians—particularly those unfamiliar with EB planning—with an intuitive visual and quantitative reference. By providing real-time visual references, the predicted dose maps assist new planners in understanding the dosimetric trade-offs, anatomical considerations, and coverage strategies specific to the EB setting. Additionally, the framework helps clarify key distinctions between EB and FB plans, illustrating how anatomical variability can impact dose distribution and supporting more consistent plan development. This enables more informed clinical decisions and fosters a deeper understanding of protocol-specific planning strategies. In multi-disciplinary settings, the model promotes a shared knowledge base across physicians, dosimetrists, and physicists, facilitating collaborative planning and communication. Ultimately, by bridging gaps in clinical experience and offering consistent, high-quality reference outputs, the model not only accelerates protocol adoption but also contributes to long-term improvements in training, quality assurance, and standardization of EB radiotherapy planning.

Beyond predictive accuracy, the proposed framework has significant clinical value. The predicted dose distributions can serve as pre-planning quality assurance (QA) references, providing radiation oncologists and dosimetrists with a dosimetrically sound benchmark early in the workflow. This additional functionality supports treatment safety, enhances plan consistency, and reduces inter-observer variability. Moreover, the visualized dose predictions can act as educational tools for clinicians adopting the EB protocol, enabling them to better understand achievable dose coverage and organ-at-risk sparing in this unique planning scenario. Furthermore, future work will focus on integrating automated dose error detection and verification mechanisms, enabling comparison between the model-predicted dose and the delivered dose distributions as an additional layer of quality assurance in clinical workflows (25, 26).

While the EB Fine-tuning model(SBRT/IMRT) demonstrated clear improvements over the General model across all evaluation metrics, it is important to acknowledge that neither model consistently outperformed the manual clinical plans as shown in **Tables 1**, **2**. These outcomes are quite expected, as manual plans are developed through an iterative, expert-driven process that incorporates patient-specific clinical considerations, physician oversight, and dynamic trade-offs between target coverage and organ-at-risk (OAR) sparing. To our knowledge, no publicly available empty bladder (EB) prostate dataset currently exists, and the ongoing RELIEF trial represents the first systematic clinical effort to establish such a dataset. The present study therefore provides an early demonstration of deep learning adaptation to the EB protocol, rather than a finalized, fully generalized model. Although the fine-tuning was performed on only 20 EB patients, this reflects the pioneering nature of the dataset rather than a methodological limitation. As the RELIEF trial expands, future work will include cross-validation and multi-institutional evaluation to strengthen model robustness. Because the data currently originates from a single institution (Mayo Clinic) within the RELIEF trial network, external validation will be necessary to ensure reproducibility across different scanners, planning systems, and institutional workflows.

Multi-institutional generalization remains a well-recognized challenge in AI-based dose prediction, primarily due to variability in contouring standards, treatment planning systems, and institutional practice. While multicenter validation is highly desirable, it is often constrained by IRB restrictions, data-sharing limitations, and TPS configuration differences. We frame this limitation as a broader field-wide challenge rather than a weakness of this study. Future collaboration through the RELIEF framework and similar international initiatives may help overcome these barriers.

In contrast to expert manual planning, deep learning models rely solely on training data and loss function optimization, without the benefit of contextual judgment or interactive feedback. A consistent underestimation of high-dose regions was observed, likely driven by the adversarial loss penalizing overestimation and by MAE-based pixel loss encouraging overly smooth gradients. Specifically, the discriminator strongly penalizes dose overshoot near the PTV edges, steering the generator toward conservative predictions. Meanwhile, the MAE loss encourages spatial averaging across steep gradients, further contributing to systematic underestimation. Clinically, this behavior tends to be safer than overshoot but may slightly reduce PTV uniformity. Future work will incorporate dose-gradient–aware loss terms, DVH-constrained optimization, and *post-hoc* calibration to reduce this systematic bias. Additionally, limitations in the training dataset—particularly the relatively small EB cohort—may constrain the model’s ability to capture the full range of anatomical variability or physician preferences reflected in clinical plans. To assess potential overfitting given the small EB cohort, we additionally performed leave-one-out cross-validation (LOOCV) across all 20 EB cases. The mean PTV Dmean error across folds was 2.31 ± 0.63 Gy and total MAPE was 3.58 ± 0.44%, indicating stable performance and suggesting that the model did not simply memorize the limited training examples. These findings reinforce the notion that while DL-based models can enhance planning efficiency, consistency, and provide high-quality starting points, they may not yet be positioned to fully replace expert manual planning. Instead, they should be viewed as decision-support tools that complement clinical expertise.

Compared with other AI-based dose prediction models (18–20), our framework is unique in explicitly targeting the EB planning scenario. By fine-tuning a cGAN on EB-specific data, this study demonstrates the first adaptation of deep learning to a protocol-specific clinical setting, establishing a foundation for future multicenter validation and cross-modality dose prediction.

Future directions include extending this methodology to proton therapy and carbon therapy, a modality that holds potential for enhanced dose conformity and superior sparing of organs at risk. Developing a dual-track workflow that integrates both photon and proton treatment modalities will enable clinicians to select the optimal approach for individual patients. Following the validation of this workflow within the RELIEF trial, we intend to implement it broadly to ensure its effectiveness and adaptability across diverse clinical settings. While the study demonstrates encouraging results, it acknowledges the limitations imposed by the relatively small size of the EB-specific dataset. Expanding this dataset through multi-institutional collaboration will enhance the model’s generalizability and robustness. Moreover, ongoing refinements to the framework, including exploring advanced architectures and loss functions, are anticipated to further improve its predictive capabilities. In addition, the predominance of IMRT plans in the FB training set (66 IMRT vs. 24 SBRT) may have biased the General model toward IMRT-like modulation patterns. This imbalance underscores the necessity of modality-specific EB fine-tuning, which corrected these patterns and restored SBRT-appropriate dose gradients.

## 5 Conclusions

This study demonstrates the feasibility of using a deep learning (DL)-based framework to predict dose distributions for prostate cancer patients treated with an empty bladder (EB) protocol. By fine-tuning a conditional generative adversarial network (cGAN) with EB-specific data from the RELIEF trial, the model achieved significantly greater accuracy compared with a general full bladder (FB)-trained model, with average errors consistently within 2.5 Gy or 3% for planning target volumes and organs at risk.

As multi-institutional EB datasets expand, the reproducibility and robustness of EB-specific dose prediction models are expected to improve further, supporting their integration into routine clinical practice. The EB Fine-tuning model(SBRT/IMRT) offers practical value beyond prediction accuracy. It provides clinically relevant reference dose distributions early in the planning workflow, functioning as both a quality assurance tool and an educational resource for EB-specific planning. By offering a consistent dosimetric benchmark, the model may improve treatment safety, plan reproducibility, and workflow efficiency as EB protocols gain wider adoption.

Overall, this work highlights the potential of DL-based approaches to streamline radiotherapy planning, improve patient-centered care, and serve as a foundation for future applications in proton and carbon ion therapy.

## Data Availability

The datasets generated and analyzed in this study include patient information and are subject to ethical restrictions. A de-identified subset of the data may be made available upon reasonable request to the corresponding author, contingent on Institutional Review Board (IRB) approval and data-sharing agreements. Requests to access the datasets should be directed to Shrestha.Deepak@mayo.edu.

## References

[1] Pinkawa M Asadpour B Gagel B Piroth MD Holy R Eble MJ . Prostate position variability and dose–volume histograms in radiotherapy for prostate cancer with full and empty bladder. Int J Radiat Oncol Biol Phys. (2006) 64:856–61. doi: 10.1016/j.ijrobp.2005.08.016, PMID: 16243443

[2] Fokdal L Honoré H Høyer M Meldgaard P Fode K von der Maase H . Impact of changes in bladder and rectal filling volume on organ motion and dose distribution of the bladder in radiotherapy for urinary bladder cancer. Int J Radiat Oncol Biol Phys. (2004) 59:436–44. doi: 10.1016/j.ijrobp.2003.10.039, PMID: 15145160

[3] Cowan RA McBain CA Ryder WD Wylie JP Logue JP Turner SL . Radiotherapy for muscle-invasive carcinoma of the bladder: results of a randomized trial comparing conventional whole bladder with dose-escalated partial bladder radiotherapy. Int J Radiat Oncol Biol Phys. (2004) 59:197–207. doi: 10.1016/j.ijrobp.2003.10.018, PMID: 15093917

[4] Nakamura N Shikama N Takahashi O Ito M Hashimoto M Uematsu M . Variability in bladder volumes of full bladders in definitive radiotherapy for cases of localized prostate cancer. Strahlenther Onkol. (2010) 186:637. doi: 10.1007/s00066-010-2105-6, PMID: 21069269

[5] O'Doherty UM McNair HA Norman AR Miles E Hooper S Davies M . Variability of bladder filling in patients receiving radical radiotherapy to the prostate. Radiother Oncol. (2006) 79:335–40. doi: 10.1016/j.radonc.2006.05.007, PMID: 16781790

[6] Malygina H Auerbach H Nuesken F Palm J Hecht M Dzierma Y . Full bladder, empty rectum? Revisiting a paradigm in the era of adaptive radiotherapy. Strahlenther Onkol. (2025) 201:47–56. doi: 10.1007/s00066-024-02306-7, PMID: 39470807 PMC12364992

[7] Dees-Ribbers HM Betgen A Pos FJ Witteveen T Remeijer P van Herk M . Inter- and intra-fractional bladder motion during radiotherapy for bladder cancer: a comparison of full and empty bladders. Radiother Oncol. (2014) 113:254–9. doi: 10.1016/j.radonc.2014.08.019, PMID: 25483834

[8] Burns D Rosbottom K Mitchell J . Is the bladder filling protocol for prostate cancer patients undergoing radiotherapy fit for purpose? Radiography. (2020) 26:S29. doi: 10.1016/j.radi.2019.11.071

[9] Moiseenko V Liu M Kristensen S Gelowitz G Berthelet E . Effect of bladder filling on doses to prostate and organs at risk: a treatment planning study. J Appl Clin Med Phys. (2006) 8:55–68. doi: 10.1120/jacmp.v8i1.2286, PMID: 17592448 PMC5722405

[10] Byun DJ Gorovets DJ Jacobs LM Happersett L Zhang P Pei X . Strict bladder filling and rectal emptying during prostate SBRT: Does it make a dosimetric or clinical difference? Radiat Oncol. (2020) 15:239. doi: 10.1186/s13014-020-01681-6, PMID: 33066781 PMC7565753

[11] Tsang YM Hoskin P . The impact of bladder preparation protocols on post treatment toxicity in radiotherapy for localised prostate cancer patients. Tech Innov Patient Support Radiat Oncol. (2017) 3-4:37–40. doi: 10.1016/j.tipsro.2017.10.001, PMID: 32095565 PMC7033795

[12] Choi B Olberg S Park JC Kim JS Shrestha DK Yaddanapudi S . Technical note: Progressive deep learning: An accelerated training strategy for medical image segmentation. Med Phys. (2023) 50:5075–87. doi: 10.1002/mp.16267, PMID: 36763566

[13] Choi BS Yoo SK Moon J Chung SY Oh J Baek S . Acute coronary event (ACE) prediction following breast radiotherapy by features extracted from 3D CT, dose, and cardiac structures. Med Phys. (2023) 50:6409–20. doi: 10.1002/mp.1639814, PMID: 36974390

[14] Park J Choi B Ko J Chun J Park I Lee J . Deep-learning-based automatic segmentation of head and neck organs for radiation therapy in dogs. Front Vet Sci. (2021) 8:721612. doi: 10.3389/fvets.2021.721612, PMID: 34552975 PMC8450455

[15] Yoo SK Kim TH Chun J Choi BS Kim H Yang S . Deep-learning-based automatic detection and segmentation of brain metastases with small volume for stereotactic ablative radiotherapy. Cancers. (2022) 14:2555. doi: 10.3390/cancers14102555, PMID: 35626158 PMC9139632

[16] Choi MS Choi BS Chung SY Kim N Chun J Kim YB . Clinical evaluation of atlas- and deep learning-based automatic segmentation of multiple organs and clinical target volumes for breast cancer. Radiother Oncol. (2020) 153:139–45. doi: 10.1016/j.radonc.2020.09.045, PMID: 32991916

[17] Kandalan RN Nguyen D Rezaeian NH Barragán-Montero AM Breedveld S Namuduri K . Dose prediction with deep learning for prostate cancer radiation therapy: Model adaptation to different treatment planning practices. Radiother Oncol. (2020) 153:228–35. doi: 10.1016/j.radonc.2020.10.027, PMID: 33098927 PMC7908143

[18] Nguyen D Long T Jia X Weiguo L Xuejun G Zohaib I . A feasibility study for predicting optimal radiation therapy dose distributions of prostate cancer patients from patient anatomy using deep learning. Sci Rep. (2019) 9:1076. doi: 10.1038/s41598-018-37741-x, PMID: 30705354 PMC6355802

[19] Bohara G Sadeghnejad Barkousaraie A Jiang S Nguyen D . Using deep learning to predict beam-tunable Pareto optimal dose distribution for intensity-modulated radiation therapy. Med Phys. (2020) 47:3898–912. doi: 10.1002/mp.14374, PMID: 32621789 PMC7821384

[20] Kui X Liu F Yang M Wang H Liu C Huang D . A review of dose prediction methods for tumor radiation therapy. Meta-Radiology. (2024) 2:100057. doi: 10.1016/j.metrad.2024.100057

[21] Olberg S Chun J Su Choi B Park I Kim H Kim T . Abdominal synthetic CT reconstruction with intensity projection prior for MRI-only adaptive radiotherapy. Phys Med Biol. (2021) 66. doi: 10.1088/1361-6560/ac279e, PMID: 34530421

[22] Olberg S Choi BS Park I Liang X Kim JS Deng J . Ensemble learning and personalized training for the improvement of unsupervised deep learning-based synthetic CT reconstruction. Med Phys. (2023) 50:1436–49. doi: 10.1002/mp.16087, PMID: 36336718

[23] Choi BS Beltran CJ Olberg S Liang X Lu B Tan J . Enhanced IDOL segmentation framework using personalized hyperspace learning IDOL. Med Phys. (2024) 51:8568–83. doi: 10.1002/mp.17361, PMID: 39167055

[24] Chetiyawardana G Hoskin PJ Tsang YM . The implementation of an empty bladder filling protocol for localised prostate volumetric modulated arctherapy (VMAT): early results of a single institution service evaluation. Br J Radiol. (2020) 93:1114. doi: 10.1259/bjr.20200548, PMID: 32706990 PMC7548355

[25] Gronberg MP Beadle BM Garden AS Skinner H Gay S Netherton T . Deep learning-based dose prediction for automated, individualized quality assurance of head and neck radiation therapy plans. Pract Radiat Oncol. (2023) 13:e282–91. doi: 10.1016/j.prro.2022.12.003, PMID: 36697347 PMC11232032

[26] Simon L Robert C Meyer P . Artificial intelligence for quality assurance in radiotherapy. Cancer Radiother. (2021) 25:623–6. doi: 10.1016/j.canrad.2021.06.012, PMID: 34176724

