# Supplementary figures and images for: Deep learning-based dose prediction for prostate cancer with empty bladder protocol: a framework for efficient and personalized radiotherapy planning
Source: Front Oncol. 2025 Dec 17;15:1690416. doi: 10.3389/fonc.2025.1690416 (PMC12753453; doi:10.3389/fonc.2025.1690416)

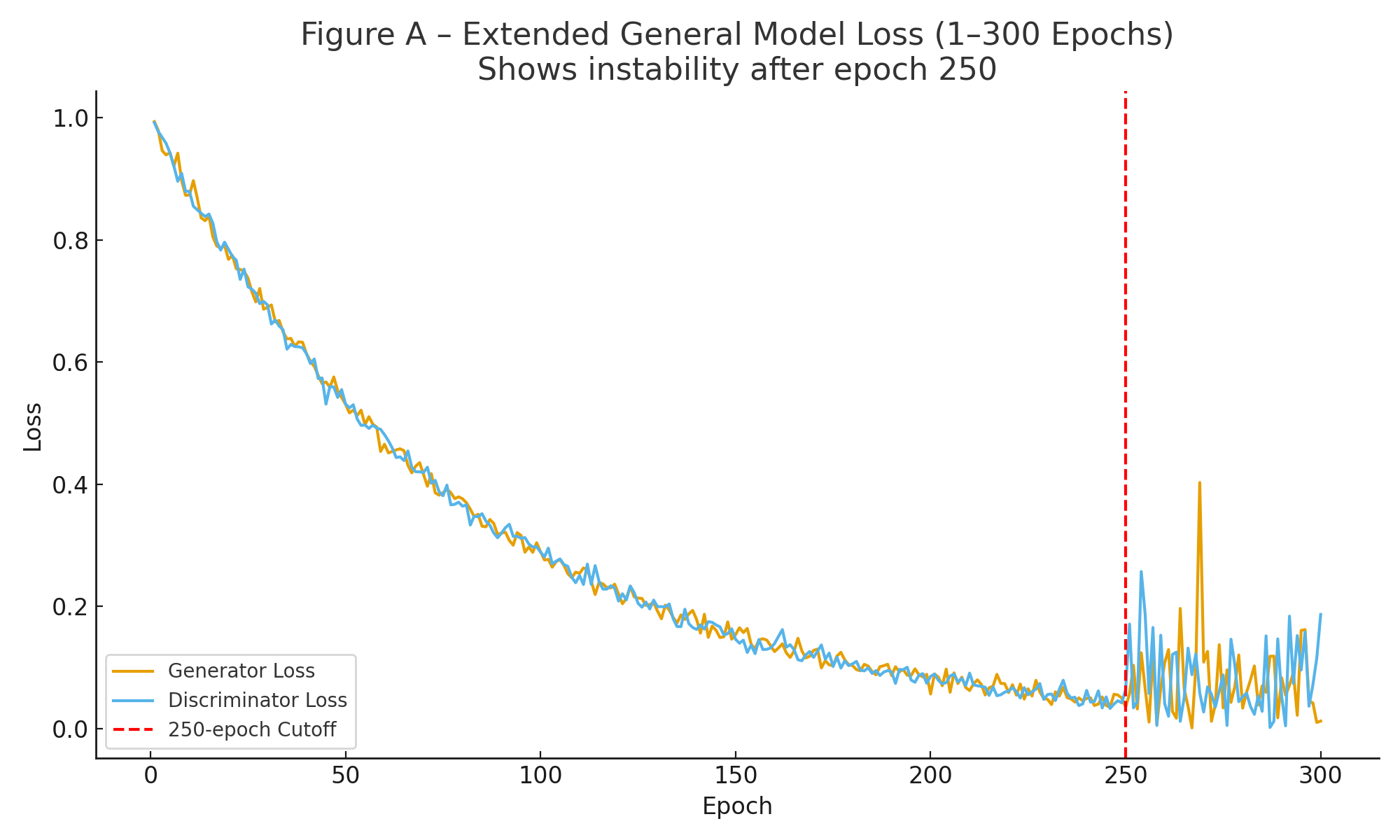

Supplement: Supplementary file 1 [file Image1.tiff]

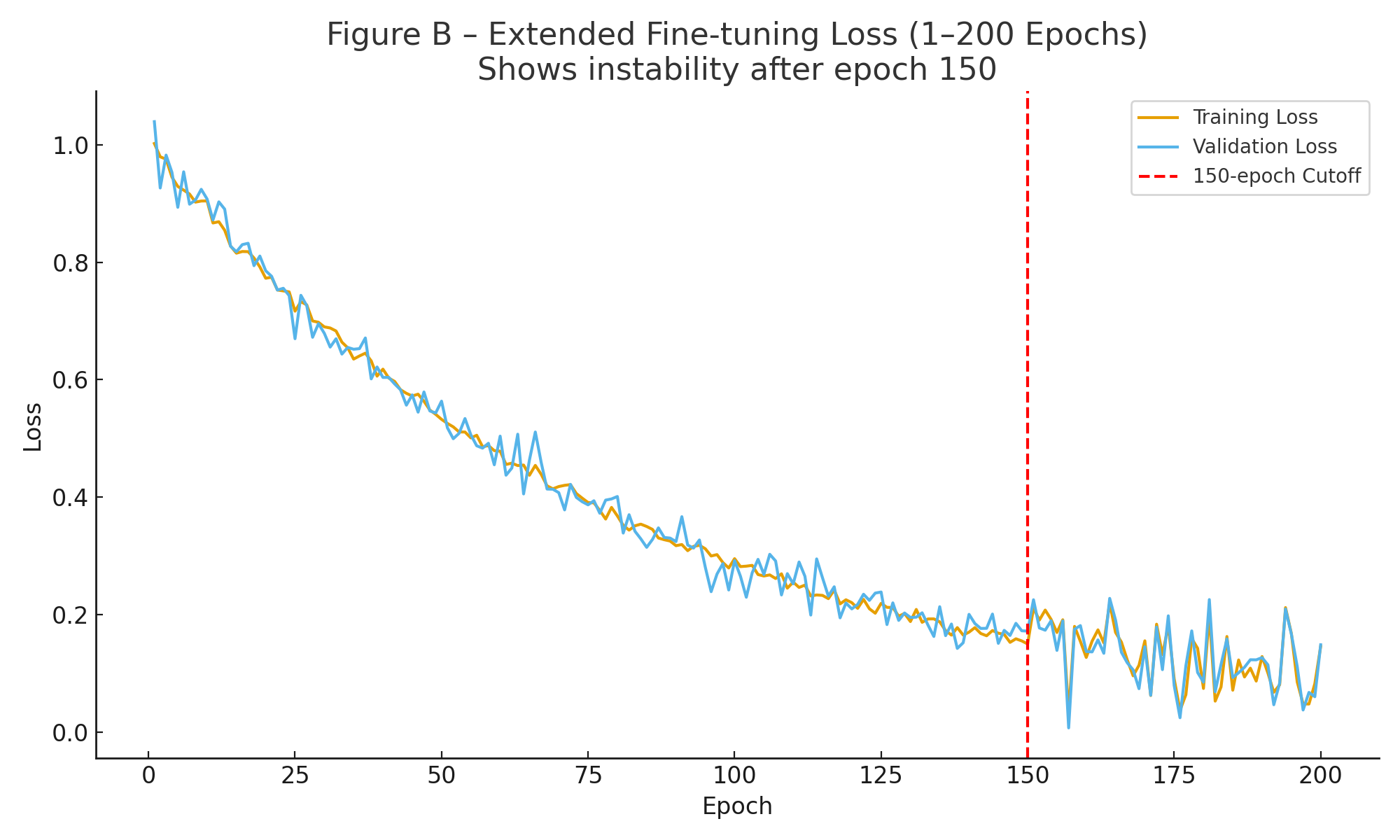

Supplement: Supplementary file 2 [file Image2.tiff]

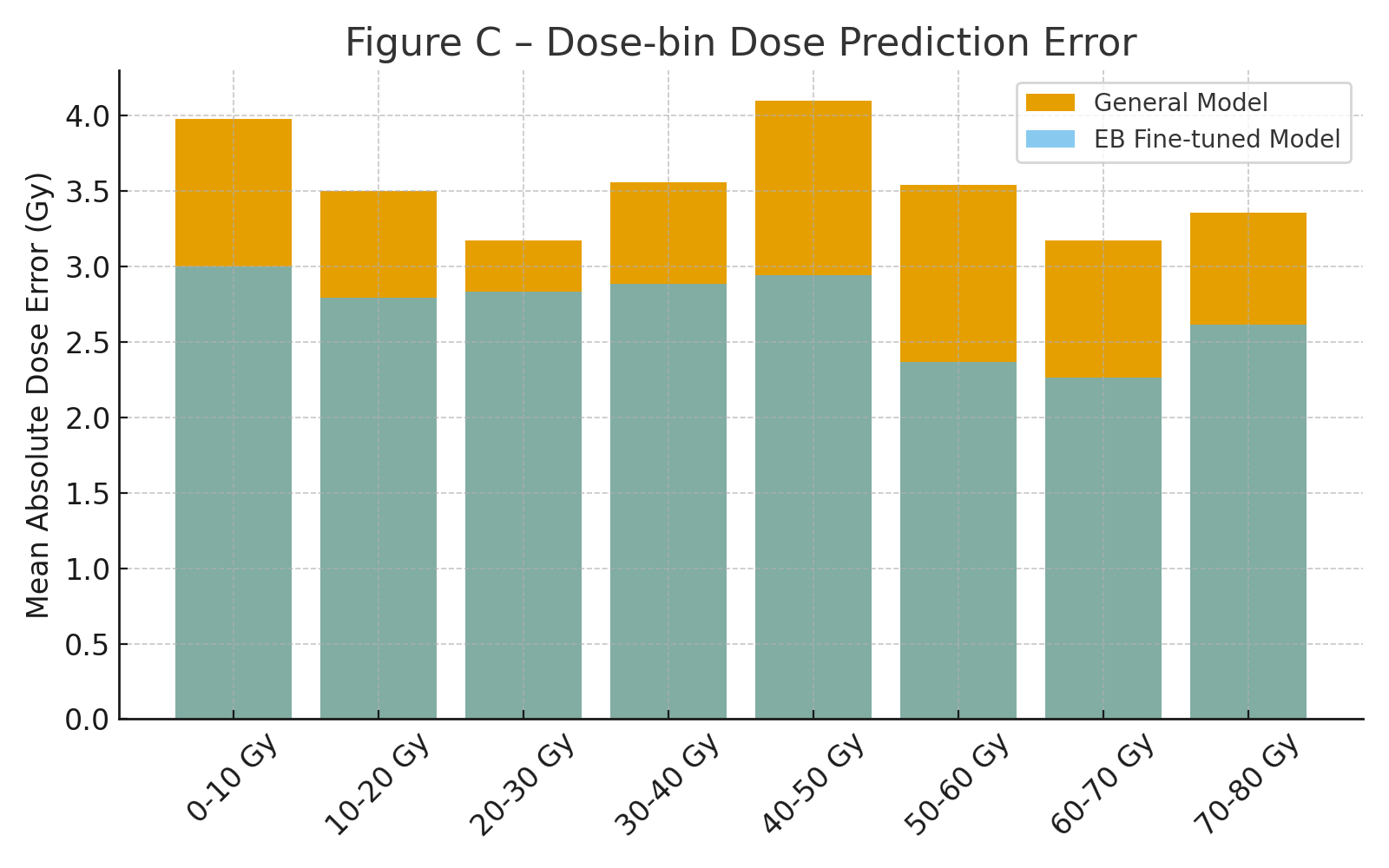

Supplement: Supplementary file 3 [file Image3.tiff]
